# Supplementary material for: From sequence to enzyme mechanism using multi-label machine learning
Source: BMC Bioinformatics. 2014 May 19;15:150. doi: 10.1186/1471-2105-15-150 (PMC4229970; doi:10.1186/1471-2105-15-150)
Supplement: Additional file 2 — Java code of ml2db. Additional file ml2db_code.tar.gz contains the Java source code to run the multi-label machine learning experiments and save the results to database. The code’s Javadoc is included. [file 1471-2105-15-150-S2.zip › additional file 2/ml2db/ml2db/doc/main/ml2db/mulan/measuremetrics/PredictionsPerformanceMeasurer.html]

PredictionsPerformanceMeasurer


JavaScript is disabled on your browser.


- Overview
- Package
- Class
- Use
- Tree
- Deprecated
- Index
- Help

- Prev Class
- Next Class

- Frames
- No Frames

- All Classes

- Summary:
- Nested |
- Field |
- Constr |
- Method

- Detail:
- Field |
- Constr |
- Method


main.ml2db.mulan.measuremetrics

## Class PredictionsPerformanceMeasurer

- java.lang.Object
- - main.ml2db.mulan.measuremetrics.PredictionsPerformanceMeasurer

- ---

    

  ```
  public class PredictionsPerformanceMeasurer
  extends java.lang.Object
  ```

  Generates prediction metrics by comparing 1. a list of predictions and . 2a
  list of true annotations.
  Takes as input a list of annotation couples (in the form of [instance, label]
  couples). One list must contain the "predicted" and the other one the
  "truth". Also a full list of the instances is needed (for the case when a set
  of predictions does not provide predictions for all the true instances).
  The predictions and true values can come from any methodology/system. For
  each instance, the class formats the predictions as Mulan MultiLabelOutput
  and the true labels as boolean[] partition.
  Generates a bunch of empty Mulan multi-label metrics (micro-averaged recall,
  subset accuracy etc.) and then, for each instance, updates each empty metric
  with the predicted and true labels. Gets the overall metric value for the set
  of instances and saves to file.

  Version:
  :   3 Dec 2011

  Author:
  :   Luna De Ferrari ldf(at)st-andrews.ac.uk

- - ### Constructor Summary

    Constructors

    Constructor and Description

    `PredictionsPerformanceMeasurer(java.lang.String instancesFilePath,
    java.lang.String predictionsFilePath,
    java.lang.String truthFilePath,
    java.lang.String regexpOfSeparator,
    java.lang.String resultsDirectoryPath)`
  - ### Method Summary

    Methods

    | Modifier and Type | Method and Description |
    | --- | --- |
    | `mulan.evaluation.Evaluation` | `fillMetrics(java.util.List<mulan.evaluation.measure.Measure> measures)` |
    | `AnnotationsReader` | `getReader()` |
    | `java.lang.String` | `getResults()` |
    | `java.lang.String` | `getResultsFilePath()` |
    | `static void` | `main(java.lang.String[] args)` |
    | `java.util.List<mulan.evaluation.measure.Measure>` | `prepareMeasures(boolean predictionHasBipartition, boolean predictionHasRanking, int numOfLabels)` |

    - ### Methods inherited from class java.lang.Object

      `equals, getClass, hashCode, notify, notifyAll, toString, wait, wait, wait`

- - ### Constructor Detail


    - #### PredictionsPerformanceMeasurer

      ```
      public PredictionsPerformanceMeasurer(java.lang.String instancesFilePath,
                                    java.lang.String predictionsFilePath,
                                    java.lang.String truthFilePath,
                                    java.lang.String regexpOfSeparator,
                                    java.lang.String resultsDirectoryPath)
      ```
  - ### Method Detail


    - #### fillMetrics

      ```
      public mulan.evaluation.Evaluation fillMetrics(java.util.List<mulan.evaluation.measure.Measure> measures)
      ```


    - #### getReader

      ```
      public AnnotationsReader getReader()
      ```

      Returns:
      :   m\_reader


    - #### getResults

      ```
      public java.lang.String getResults()
      ```

      Returns:
      :   m\_results


    - #### getResultsFilePath

      ```
      public java.lang.String getResultsFilePath()
      ```


    - #### prepareMeasures

      ```
      public java.util.List<mulan.evaluation.measure.Measure> prepareMeasures(boolean predictionHasBipartition,
                                                                     boolean predictionHasRanking,
                                                                     int numOfLabels)
      ```


    - #### main

      ```
      public static void main(java.lang.String[] args)
      ```


- Overview
- Package
- Class
- Use
- Tree
- Deprecated
- Index
- Help

- Prev Class
- Next Class

- Frames
- No Frames

- All Classes

- Summary:
- Nested |
- Field |
- Constr |
- Method

- Detail:
- Field |
- Constr |
- Method
